# Blood feeding-induced transcriptomic changes in the hard tick *Ixodes persulcatus*

**DOI:** 10.3389/finsc.2026.1669026

**Published:** 2026-02-23

**Authors:** Yihan Lou, Bin Wu, Wenwu Yao, Chuanxi Zhang, Haijun Xu, X. Frank Yang, Xuechao Zhang, Zhangnv Yang

**Affiliations:** 1 Department of Microbiology, Zhejiang Provincial Center for Disease Control and Prevention, Hangzhou, China; 2 Department of Testing and Inspection, Jinhua Center for Disease Control and Prevention, Jinhua, Zhejiang, China; 3 Institute of Insect Sciences, Zhejiang University, Hangzhou, China; 4 Department of Microbiology and Immunology, Indiana University School of Medicine, Indianapolis, IN, United States; 5 Department of Expanded Program on Immunization, Hangzhou Center for Disease Control and Prevention, Hangzhou, China

**Keywords:** antioxidant system, blood meal, *Ixodes persulcatus*, *Leishmania*, metabolic detoxification system, transcriptome

## Abstract

**Introduction:**

Ticks are hematophagous ectoparasites that must overcome significant physiological challenges during blood feeding. These include managing oxidative stress, detoxifying host-derived molecules, and reallocating energy to support digestion, tissue remodeling, and reproduction.

**Methods:**

In this study, we conducted a *de novo* transcriptome assembly and genome-wide transcriptional profiling of female *Ixodes persulcatus* ticks at three key feeding stages: unfed, semi-engorged, and fully engorged. Functional annotation and Gene Ontology (GO) enrichment analyses were conducted to characterize stage-associated transcriptional changes, with a focus on metabolic detoxification and antioxidant systems.

**Results and discussion:**

We generated a reference transcriptome containing 56,900 unigenes. Comprehensive analyses of metabolic detoxification and antioxidant systems revealed species-specific expansions in key supergene families such as cytochrome P450s and glutathione S-transferases. The expression profiles across feeding stages revealed pronounced physiological changes in response to blood meal, and GO enrichment analysis showed that these changes were mainly involved in blood acquisition, nutrient metabolism, respiratory processes, hormone synthesis, egg development, immune responses, ROS detoxification, transcription and translation. These findings offer new insights into the molecular physiology of tick hematophagy and provide a valuable resource for future studies on stress responses and metabolic regulation in ticks.

## 1 Introduction

Ticks are globally recognized as critical vectors for a wide range of pathogenic microorganisms, including viruses, bacteria, and protozoa. Many of these pathogens pose significant threats to public health and livestock production (1, 2), such as *Borrelia burgdorferi* (Lyme disease), *Babesia* spp. (babesiosis), and tick-borne encephalitis virus (3–5). These wide-ranging health and economic impacts are tightly linked to the tick’s unique hematophagous lifestyle. Unlike many blood-feeding arthropods, hard ticks (*Ixodidae*) undergo prolonged attachment to their hosts, ingesting large volumes of blood—often over 100 times their body weight—which facilitates efficient pathogen transmission and exposes them to significant physiological stress.

Blood feeding is essential for tick development, with one meal required at each life stage to trigger molting or oviposition (6). To overcome host defenses during prolonged feeding, ticks secrete pharmacologically active saliva to inhibit coagulation, modulate immune responses, and suppress inflammation (7). Beyond countering host defenses, ticks must also cope with internal oxidative stress arising from blood ingestion—especially from the accumulation of heme, free iron, and other pro-oxidant molecules that can damage cellular components (8). To maintain cellular homeostasis, ticks rely on detoxification and antioxidant systems, including glutathione S-transferases (GSTs), cytochrome P450 monooxygenases (CYPs), superoxide dismutases (SODs), catalases (CAT), and glutathione peroxidases (GPx) (9).

The tick midgut plays a central role in nutrient acquisition, digestion, and physiological adaptation during blood feeding, serving as the primary site for erythrocyte lysis and hemoglobin degradation—processes that expose the tissue to high concentrations of heme and free iron, two potent inducers of reactive oxygen species (ROS) that can damage cellular membranes, proteins, and DNA (10, 11). Ticks do not excrete excess heme but instead sequester and detoxify it intracellularly, often within hemosomes in midgut epithelial cells - a strategy that places the midgut at the forefront of redox regulation and metabolic stress management (12). Transcriptomic and functional studies have confirmed the expression of multiple antioxidant and detoxification enzymes in tick midguts, supporting their critical role in maintaining redox balance and processing host-derived toxins during feeding (11, 13, 14).

The advent of next-generation sequencing (NGS) technologies has significantly advanced the study of tick biology by enabling high-resolution transcriptomic profiling across tissues, developmental stages, and feeding timepoints. These studies have uncovered complex gene expression programs in key organs such as the salivary glands, ovaries, and midguts of *Ixodes ricinus*, *Rhipicephalus microplus*, and *Amblyomma sculptum*, with functional links to hematophagy, immune regulation, and nutrient metabolism (15–17). In particular, midgut-specific transcriptome analyses have revealed dynamic shifts in gene expression in response to feeding progression, highlighting the tightly regulated oxidative and metabolic processes associated with blood digestion (18, 19). Nonetheless, transcriptomic resources for many ecologically important tick species remain scarce, and studies focusing specifically on the detoxification and antioxidant gene families in the midgut are limited.

*Ixodes persulcatus*, a dominant vector species in Eurasia, is well adapted to cold temperate habitats and transmits multiple zoonotic pathogens. However, little is known about its molecular responses to oxidative and metabolic stress during blood feeding. In this study, we conducted a *de novo* transcriptome assembly of female *Ixodes persulcatus* and analyzed gene expression profiles across three feeding stages. Our primary focus was on the transcriptional dynamics of metabolic detoxification and antioxidant gene families, aiming to better understand their roles in physiological adaptation during hematophagy. Although further validation is needed, this finding underscores the value of transcriptomic approaches in revealing novel molecular mechanisms underlying tick adaptation and physiology. Together, our findings provide new insights into the molecular basis of blood-feeding adaptation and lay a foundation for further functional investigation of stress-response mechanisms in ticks.

## 2 Materials and methods

### 2.1 Tick preparation

The *I. persulcatus* ticks used in this study were collected from woodlands near Mudanjiang (N 44.916°, E 129.498°) in May 2012. This region is known as an endemic area for Lyme borreliosis and anaplasmosis (20, 21). Field-collected ticks were maintained in conditional incubators. Adult female ticks were allowed to feed on naïve Sprague Dawley rats according to the protocols approved by the Institutional Animal Care and Use Committee of Zhejiang University.

To ensure stage consistency, unfed, semi-engorged, and fully engorged ticks were selected based on both feeding duration and morphological characteristics. Semi-engorged ticks were collected five days post-attachment, corresponding to the late slow-feeding phase. Fully engorged ticks were defined as those that had voluntarily detached from the host after completing blood feeding and displayed a fully distended, spherical body typical of replete females 7 days post attachment. All ticks were surface sterilized in 70% ethanol via gently shaking for 30 s and then rinsed three times in sterile water to remove environmental contaminants before total RNA extraction.

### 2.2 Illumina library preparation, sequencing and data analysis

Each pooled sample of unfed, semi-engorged and fully engorged female ticks was pulverized thoroughly in liquid nitrogen and then subjected to total RNA extraction using RNAiso Plus (TaKaRa, Dalian, China). The extracted RNA was then treated with DNase to remove any genomic contamination. RNA quality and integrity were assessed using the Agilent 2100 Bioanalyzer (Agilent Technologies, USA). All replicative samples had RNA integrity number (RIN) values ≥ 8.0, indicating high-quality RNA suitable for sequencing. To obtain an integrated gene expression information, the RNA samples were pooled at equimolar concentrations for Illumina sequencing.

The cDNA library was constructed in accordance with the Illumina manufacturer’s instructions. Briefly, the purified mRNA, using oligo(dT) magnetic beads, was interrupted into short fragments in the presence of divalent cations at 94°C for 5 min. The fragmented RNA was then used for double strand cDNA synthesis, followed by end-repair and ligation of adaptors. To select the appropriately sized fragments, the products were separated on agarose gels. After purification using a QIAquick PCR purification Kit (QIAGEN, China), the cDNA library was enriched by PCR amplification.

High-throughput sequencing of the cDNA library was performed on the Illumina Hiseq2000 sequencing system in paired-end run (100bp) (BGI, ShenZhen, China). To eliminate low-quality reads, the raw reads transformed from the raw Illumina image data were trimmed as follows: reads only with adapters, those with more than 5% unknown nucleotides, and those of low quality (≥ 20% of the bases with a quality score (Q) ≤ 10). Then, *de novo* assemblies were performed using the Trinity software package (22), and the assembled sequences were clustered using the TGICL Clustering tool (23) and Phrap assembler to generate unigenes, which were subsequently annotated using the blastx algorithm against the nr, Swiss-Port, KEGG, and COG databases (as of 2025-09-05). For further annotation of the unigenes, GO annotation analysis was carried out using the Blast2GO program (24), and the GO terms were subsequently categorized using the WEGO software (25). The Blast2GO program, which is based on the Kyoto Encyclopedia of Genes and Genomes (KEGG), was also used to predict the pathways in which the putative peptides of the unigenes might participate.

### 2.3 Orthologous gene family and phylogenetic analyses

Phylogenetic analysis was performed based on protein sequences of 8 species, including Ixodes persulcatus (https://bigd.big.ac.cn/gwh/Assembly/8896/show ), *Ixodes scapularis* (GCF_016920785.2), *Dermacentor silvarum* (https://bigd.big.ac.cn/gwh/Assembly/8869/show/), *Dermacentor variabilis* (GCF_050947875.1), *Haemaphysalis longicornis* (https://bigd.big.ac.cn/gwh/Assembly/8865/show), *Hyalomma asiaticum* (https://bigd.big.ac.cn/gwh/Assembly/8867/show), *Rhipicephalus sanguineus* (https://bigd.big.ac.cn/gwh/Assembly/8868/show), and *Aedes aegypti* (GCF_002204515.2). For each species, only the longest protein isoform per gene was retained to represent the corresponding coding gene.

OrthoFinder v2.5.5 with default parameters was used to determine the orthologs and paralogs. All identified 368 single-copy orthologs were aligned using MAFFT v7.520 and trimmed utilizing trimAl v1.4.1 The maximum likelihood (ML) tree was constructed using IQ-TREE v2.2.3with 1,000 ultrafast bootstrap replicates, and *A. aegypti* was used as an outgroup. Divergence time were calibrated using TIMETREE database and a divergence time tree was generated with MCMCTree package in PAML v4.10.7 For identifying the gene family expansion and contraction, CAFE v5 with Viterbi p-value < 0.05 was used to examine gene family clusters.

To visualize the overlap of gene families among the eight species, the OrthoFinder orthogroup table was converted into a binary presence/absence matrix in Python (Pandas v1.2.4), in which each orthogroup was coded as “1” or “0” in a given species. An UpSet plot was then generated using the upsetplot package v0.7.0 to summarize shared and lineage-specific gene family intersections.

### 2.4 Quantitative real-time PCR validation of RNA-seq data

To validate the RNA-seq results, quantitative real-time PCR (qRT-PCR) was performed on a subset of 16 unigenes, together with the reference gene *RPS14*. Total RNA was extracted as described above RNA purity and concentration were assessed with a NanoDrop 2000 spectrophotometer (Thermo Fisher Scientific, Bremen, Germany). First-strand cDNA was synthesized from 1μg of total RNA using the ReverTra Ace^®^ qPCR RT Master Mix with gDNA Remover (ToYoBo, Osaka, Japan). Gene-specific primers for 16 selected unigenes were designed using Primer Premier 6.0 software (PREMIER Biosoft, Palo Alto, CA, USA). Quantitative real-time PCR was performed using the CFX96™ Real-Time PCR Detection System (Bio-Rad, Hercules, CA, USA) under the following thermal cycling conditions: initial denaturation at 95°C for 3min, followed by 40 cycles of 95°C for 10s and 60°C for 30s. Each biological group consisted of three independent biological replicates, and each reaction was conducted in technical triplicate.

The housekeeping gene *RPS14* was used as a reference gene to normalize gene expression levels. RNA-seq data showed low variation in RPS14 transcript abundance across unfed, semi-engorged and fully engorged ticks (**Supplementary Table S1**). In addition, qRT-PCR analysis confirmed that *RPS14* Ct values remained stable between the fed and unfed ticks (**Supplementary Table S1**). These results support the use of *RPS14* as a suitable reference gene for relative quantification. For *RPS14*, amplification efficiency was evaluated using a standard curve generated from a three-point 10-fold dilution series. The Ct versus log10(concentration) plot yielded a slope of −3.54 (R^2^ = 0.997), corresponding to an amplification efficiency of approximately 92% (**Supplementary Figure S1**). All primer pairs used in this study produced single-peak melting curves, indicating high specificity of the assays. Relative gene expression levels were calculated using the 2^–ΔΔCt^ method. The expression patterns obtained by RT-qPCR were compared to those observed in RNA-seq to assess the consistency and reliability of transcriptome data.

### 2.5 Identification of *I. persulcatus* metabolic detoxification gene families

The tblastn program was used to identify metabolic detoxification proteins in the *I. persulcatus* transcriptome using the nr database with the published metabolic detoxification enzymes of *D. melanogaster*, *A. gambiae*, *Apis mellifera* and *Tetranychus urticae* as reference datasets (26–30). The initial candidate sequences were confirmed by identifying the conserved domains (CDD, http://www.ncbi.nlm.nih.gov/cdd), and were manually selected to exclude redundant sequences. Considering that most genes encoding cytochrome P450 monooxygenases (P450s) and carboxylesterases (CarEs) were partially sequenced and may be different parts of the same genes, the remaining sequences were mapped to the *I. scapularis* sequences deposited in the NCBI database using the blastn program. Once the sequences were mapped to the same blast results with high homology, the longer sequence was adopted. Subsequently, we assigned each candidate sequence to specific superfamilies based on the best blast hits of the reference data set instead of by using phylogenetic analysis in the initial analysis. For glutathione *S*-transferases (GSTs), the candidate sequences were further confirmed by identifying the conserved residues of the catalytic sites. Only the sequences with intact domains and catalytic sites were used for further analyses. To infer the phylogenetic relationships of the GST genes, the protein alignments were performed using the Clustal X software (version 2.0), with default gap penalties, and phylogenetic trees were constructed using the maximum likelihood method implemented in MEGA6 (31).

### 2.6 Annotation of *I. persulcatus* antioxidant genes

In order to find the candidate antioxidant genes of *I. persulcatus* and *I. scapularis*, the published antioxidant genes of *D. melanogaster*, *A. gambiae* and *A. mellifera* were used as reference datasets (32, 33). Searches were performed using the tblastn program against the *I. persulcatus* transcriptome or using the blastp program against the *I. scapularis* genome at VectorBase (https://www.vectorbase.org/). The candidate antioxidant genes were further confirmed by identifying the conserved residues of catalytic sites and the conserved domains (CDD, http://www.ncbi.nlm.nih.gov/cdd). Only the sequences with intact domains and catalytic sites were used for further analysis. The phylogenetic trees were constructed according to the method mentioned above for inferring the phylogenetic relationship among the candidate antioxidant genes. Then the *I. scapularis* and *I. persulcatus* antioxidant gens were named following the norms described previously (32, 33).

### 2.7 RNA-seq libraries of ticks at different feeding phases

For each feeding stage (unfed, semi-engorged, and fully engorged), total RNA was extracted from a pool of 10 female adult ticks to construct a single RNA-seq library. Three biological replicates were included in this study. RNA extraction and library construction were performed as described above. High-throughput sequencing of the RNA-seq libraries were carried out on the Illumina Hiseq2000 sequencing system in paired-end run (100bp) (BGI, Shenzhen, China).

To obtain clean reads, the raw data were filtered by removing read adapters, those with more than 4% unknown nucleotides, and those of low quality (≥ 50% of the bases with a quality score (Q) ≤ 20) and only the reads ≥20 bp were retained. After this step, SOAPalinger/soap2 (34) was used to align the Illumina reads against our transcriptome reference database, allowing no more than two mismatches. Subsequently, transcript abundances were quantified by FPKM (35) and DEGseq program (36) was implemented to analyze gene differential expression between conditions. The false discovery rate (FDR) was used to determine the threshold p-value in multiple-testing correction. We selected absolute value of log2 >1 and FDR <0.05 as a threshold to define the differentially expressed genes. GO and KO enrichment analysis of the differentially expressed genes were performed by fisher hypergeometric test and FDR < 0.05 was selected as a threshold to determine significantly enriched gene sets.

### 2.8 Availability of supporting data

The data sets support the results of this article have been deposited in the NCBI Transcriptome Shotgun Assembly (TSA) database under the accession PRJNA263101 and in the NCBI Short Read Archive (SRA) database under the accession SRP048637.

## 3 Results

### 3.1 Transcriptome sequencing and validation

**Figure 1:**
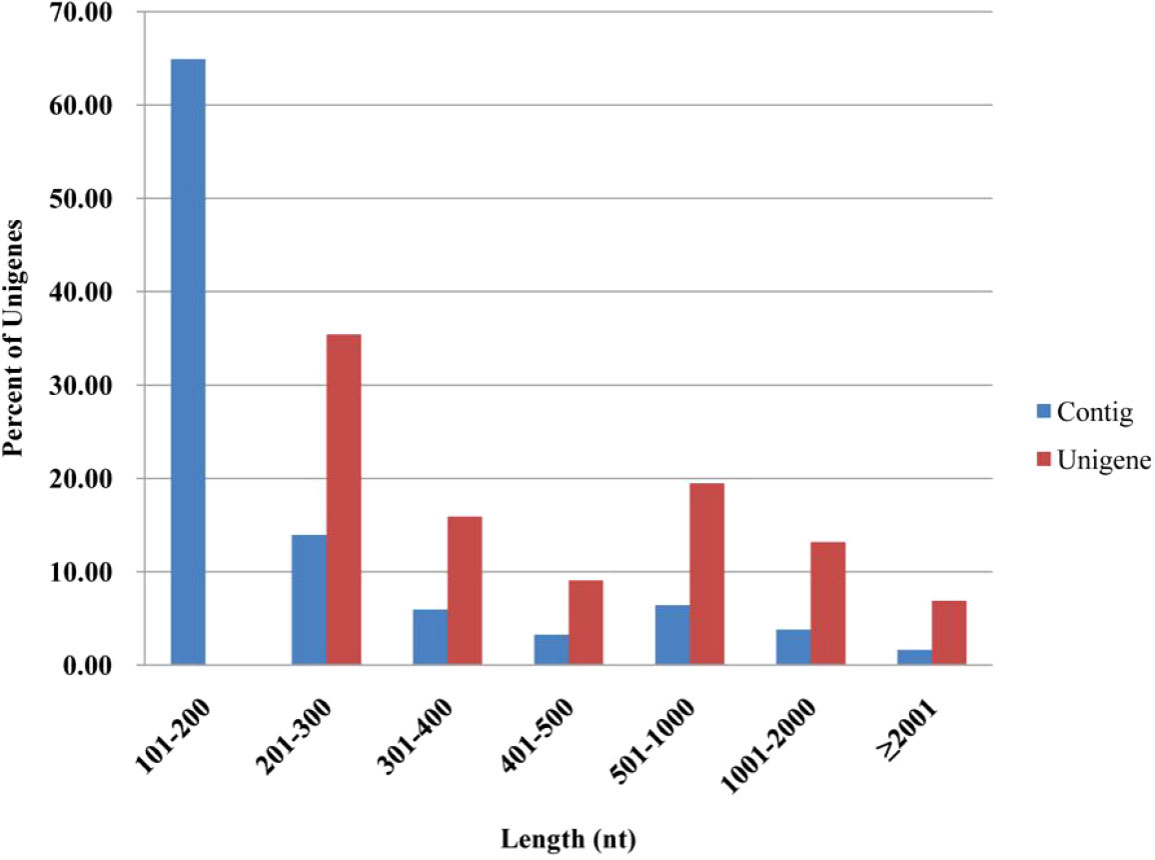
The size distribution of contigs and unigenes.

Illumina Hiseq2000 sequencing of pooled *I. persulcatus* female ticks yielded a total of 57,318,460 reads. After removing low-quality reads, the remaining 53,579,464 clean reads were assembled into 147,346 contigs (N50 = 480 nt). To reduce redundant sequences, the TGICL Clustering tool and Phrap assembler were implemented and generated 56,900 unigenes (N50 = 1,164 nt), which comprised of 14,478 distinct clusters and 42,422 distinct singletons. The size distribution analysis indicated that all of the unigenes were longer than 200 nt and that more than 20% of the unigenes (11,421 unigenes) were longer than 1,000 nt (**Figure 1**). These unigenes were used as the reference transcriptome in this study. These data provide a comprehensive resource for further understanding the physiological events of *I. persulcatus* at the molecular level.

To validate the accuracy of RNA-seq data, we performed RT-qPCR analysis on 16 selected unigenes exhibiting a range of up- and down-regulated expression patterns. The expression trends observed by RT-qPCR were generally consistent with the RNA-seq results (**Supplementary Figure S2** and **Supplementary Table S2**). Correlation analysis between RT-qPCR (−ΔΔCq) and RNA-seq (log_2_ fold change) values revealed a strong positive correlation (Pearson’s r = 0.962, p < 0.0001), indicating the high reliability of the transcriptomic data.

### 3.2 Comparative genomic characterization of *I. persulcatus*

**Figure 2:**
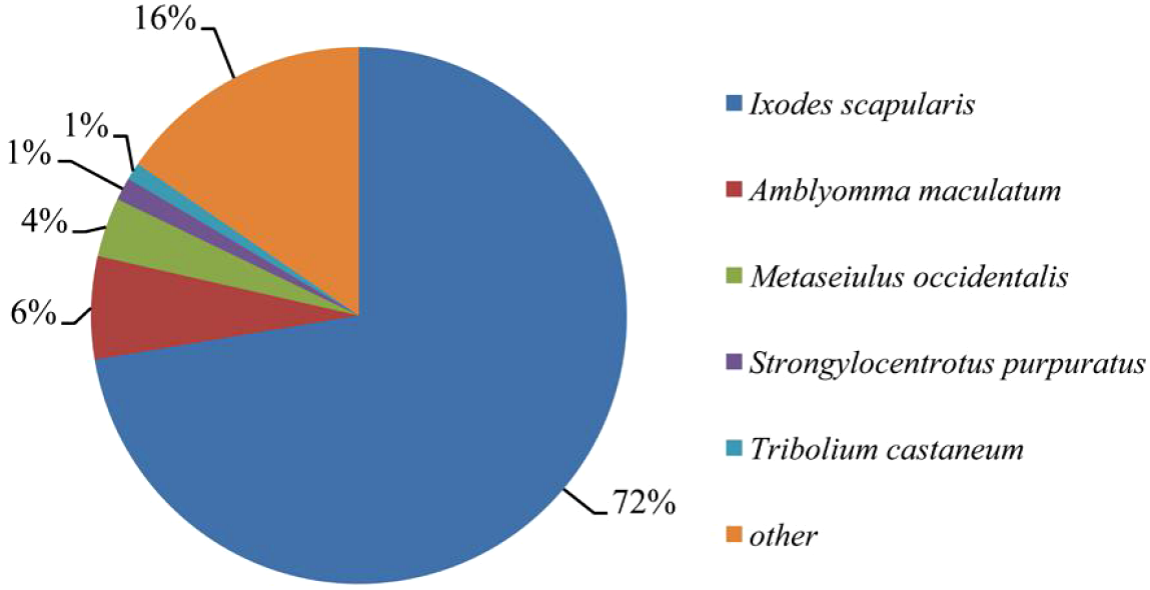
Species distribution of the top blastx hits of the *I. persulcatus* unigenes against nr database. Different colors represent different species, and species with relative abundance less than 1% was assigned as ‘other’.

Approximately 46% (26,262/56,900) of the *I. persulcatus* unigenes were annotated as sequences with significant similarity to those deposited in the GenBank nr database (E-value <10^-5^); the remaining 54% of the unigenes were not annotated. Of the annotated unigenes, the vast majority (72%) mapped to the *I. scapularis* tick genome (NCBI RefSeq assembly: GCF_016920785.2), largely due to their phylogenetic relatedness. The remaining unigenes matched to the genomes of *Amblyomma maculatum* (6%), *Metaseiulus occidentalis* (4%), *Strongylocentrotus purpuratus* (1%), and *Tribolium castaneum* (1%) (**Figure 2**). Ticks have divergent evolutionary histories exceeding a hundred million years, and the numerous niches for ticks have made the lineages highly divergent (37, 38). In contrast to the extensive genome resources of insects, high-quality genome assemblies for ticks remain scarce (39). Thus, these reasons may account for more than half of the proportion of *I. persulcatus* unigenes that lack homology to the existing database.

**Figure 3:**
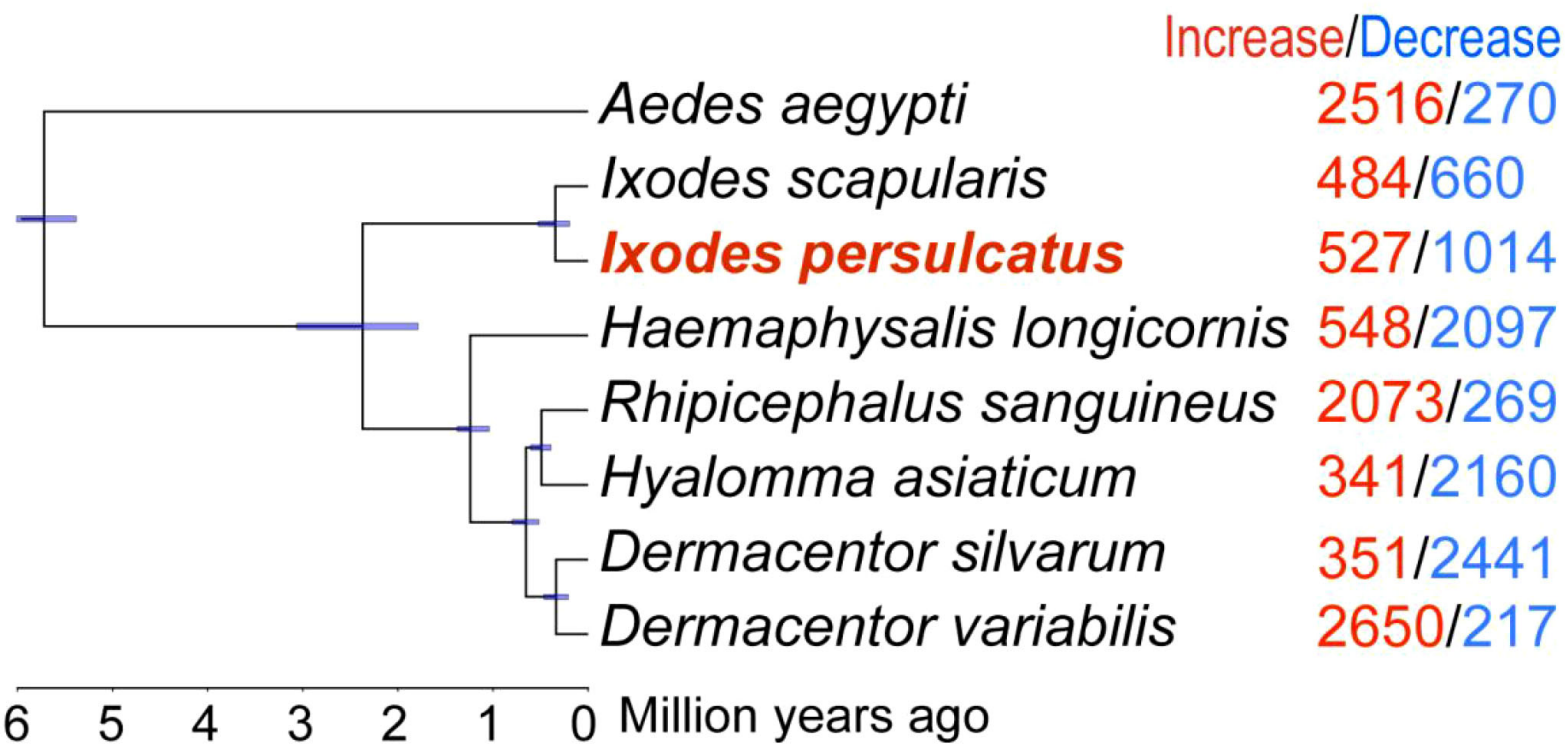
Phylogenetic analysis. A maximum likelihood (ML) tree based on *I. persulcatus* and 7 other species. All nodes are supported by 1,000 bootstrap replicates. The numbers of expanded gene families (red) and contracted gene families (blue) are shown on the right of each branches. The scale at the bottom represents the divergence time. The purple bars beside the nodes indicate the divergence time range with 95% confidence intervals.

To place the *I. persulcatus* transcriptome in a broader tick genomic context, we next performed an ortholog-based comparative analysis including seven tick species and one mosquito outgroup with available genome assemblies: *I. persulcatus*, *I. scapularis*, *Dermacentor silvarum*, *Dermacentor variabilis*, *Haemaphysalis longicornis*, *Hyalomma asiaticum*, *Rhipicephalus sanguineus*, and *Aedes aegypti*. Using OrthoFinder, we identified 368 single-copy orthologous genes shared by all eight species and used these to reconstruct a maximum-likelihood species tree and estimate divergence times (**Figure 3**). The resulting topology is congruent with current tick systematics, with *I. persulcatus* forming a well-supported clade with *I. scapularis* within the genus *Ixodes*. Divergence time estimates indicate that the split between *I. persulcatus* and *I. scapularis* is relatively recent compared with deeper separations among Prostriata and Metastriata ticks.

**Figure 4:**
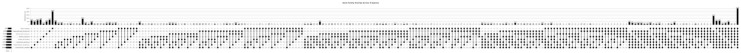
Gene family overlap across 8 species. The UpSet plot shows the overlap of gene families across 8 species, highlighting the shared and unique gene families. Each horizontal bar represents a specific gene family, and the height of the bar indicates the number of species in which the gene family is present. The intersections show the overlap of gene families between multiple species.

To further examine patterns of shared and unique gene families among species, we generated an UpSet plot summarizing orthogroup presence and absence across the eight genomes (**Figure 4**). The largest intersection corresponded to core gene families present in all eight species, likely representing fundamental arthropod housekeeping functions. Among tick-only intersections, prominent sets comprised gene families shared by all seven tick species, as well as families shared specifically by *I. persulcatus* and *I. scapularis*, reflecting both tick-wide and genus-level conservation. In addition, a subset of orthogroups was uniquely present in *I. persulcatus*, highlighting species-specific innovations that may contribute to its ecological specialization and vector competence.

### 3.3 GO and KEGG annotation

**Figure 5:**
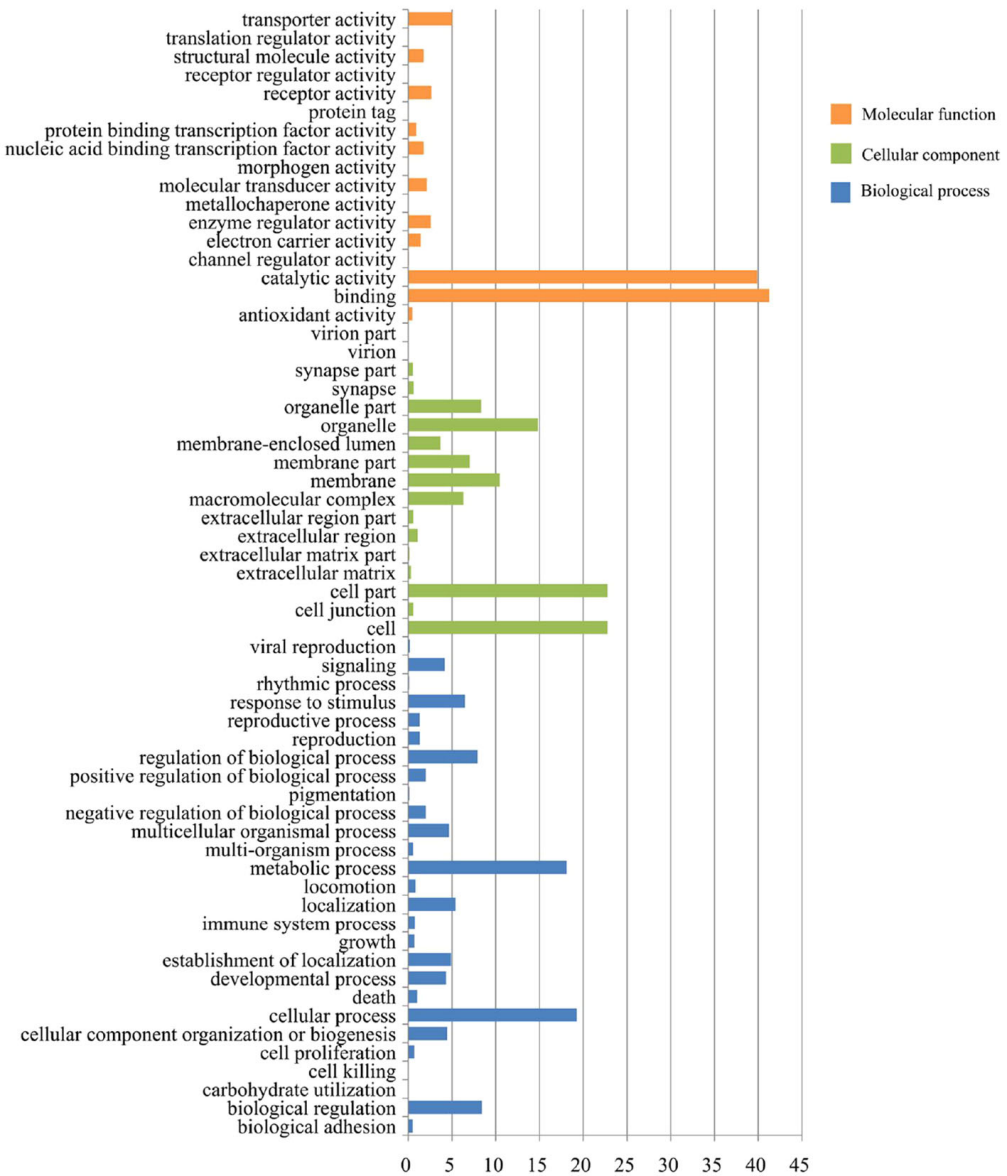
Histogram presentation of Gene Ontology classifications for the *I. persulcatus* unigenes.

Sequence analyses assigned 15,702 unigenes into one or more GO terms (**Supplementary Table S3**). These annotated unigenes were classified into biological processes (10,291 unigenes, 68.3%), cellular components (9,098 unigenes, 57.9%) and molecular functions (13,260 unigenes, 88.5%), and were involved in a wide range of functional categories (**Figure 5** and **Supplementary Table S3**). Within the biological process group, cellular processes (19.2%) and metabolic processes (18.1%) were found to be the predominant functional categories, followed by biological regulation (8.4%), regulation of biological processes (7.9%), and response to stimulus (6.5%), indicating that a considerable number of tick genes were involved in response to environment stimuli during blood feeding. For the molecular function group, a majority of unigenes were involved in binding (41.3%) and catalytic activity (39.9%). For the cellular component group, almost half of the unigenes were highly prevalent in cell (22.8%) and cell parts (22.8%).

For a comprehensive understanding of the specific responses and functions involved in blood feeding in *I. persulcatus* ticks, the unigenes were submitted to KEGG analysis to determine the putative pathways. The results showed that 17,353 unigenes of the *I. persulcatus* tick were annotated to 256 KEGG pathways. The major pathways represented by the unigenes (*n*>500) include metabolic pathways (*n* = 2,418), amebiasis (*n* = 937), focal adhesion (*n* = 816), RNA transport (*n* = 728), and protein digestion and absorption (*n* = 592) (**Supplementary Table S4**).

### 3.4 Metabolic detoxification enzymes of *I. persulcatus*

We systematically analyzed three major metabolic detoxification enzyme families: cytochrome P450 monooxygenases (P450s), glutathione S-transferases (GSTs), and carboxylesterases (CarEs) in *I. persulcatus*. These enzyme families are potentially relevant not only to acaricide resistance but also to broader physiological responses of ticks to blood feeding and pathogen exposure.

We identified 166 P450-related unigenes in the *I. persulcatus* transcriptome. These unigenes were then assigned to four clans: 38 to CYP2, 80 to CYP3, 44 to CYP4 and 4 to the mitochondrial clan (CYP M) (**Table 1** and **Supplementary Table S5**). Notably, expansions were observed in the CYP2 and CYP3 clans relative to insects, as shown in **Table 1**. A total of 33 GST genes were found in the *I. persulcatus* transcriptome, a number comparable to that found in *I. scapularis* tick (**Table 1** and **Supplementary Table S6**). Phylogenetic analysis revealed that the GST supergene family of *I. persulcatus* was similar to that of its phylogenetically related species, *I. scapularis* (**Supplementary Figure S3**). Briefly, 31 GST genes were classed into five different cytosolic GST classes: Delta (7), Epsilon (3), Mu (14), Omega (3), and Zeta (4). In addition, we identified one mitochondrial Kappa class GST gene and one microsomal GST gene in the transcriptome database. The Mu and Delta classes suffered expansions in relation to some insects (**Table 1**). We also identified 62 CarE-related unigenes, of which 49 were unique (**Supplementary Table S7**). Functional classification of these CarEs showed that 2 unigenes were associated with dietary/detoxification functions, 5 with hormone/semiochemical processing, and 42 with neuro/development (**Table 1** and **Supplementary Table S7**).

**Table 1:** Overview of metabolic detoxification enzymes in Insecta and Arachnida.

Class species	Insecta							Arachnida		
	Dmel	Agam	Aaeg	Amel	Nvit	Tcas	Bmor	Turt	Iper	Isca
Cytosolic GSTs										
delta	11	12	8	1	5	3	4	16	7	7
epsilon	14	8	8	–	–	19	8	–	3	5
mu	–	–	–	–	–	–	–	12	14	14
omega	5	1	1	1	2	4	4	2	3	3
sigma	1	1	1	4	8	7	2	–	–	–
theta	4	2	4	1	3	1	1	1	–	–
zeta	2	1	1	1	1	1	2	–	4	3
unknown	–	3	3	–	–	–	2	–	–	–
Cytosolic GST total	37	28	27	8	19	35	23	31	31	32
P450s										
CYP2	7	10	12	8	7	8	7	48	38	39
CYP3	36	40	82	28	48	72	30	10	80	120
CYP4	32	46	57	4	30	45	36	23	44	46
CYPM	11	9	9	6	7	9	12	5	4	–
P450 total	88	105	160	46	92	134	85	86	166	205
CarEs										
Dietary class	13	16	22	8	13	26	57	–	2	–
Hormone/semiochemical	8	14	14	5	17	11	8	2	5	8
Neuro/development	14	21	18	11	11	12	11	69	42	30
CarE total	35	51	54	24	41	49	76	71	49	38

Dmel, D. melanogaster; Agam, A. gambiae; Aaeg, A. aegypti; Amel, A. mellifera; Nvit, Nasonia vitripennis; Tcas, T. castaneum; Bmor, Bombyx mori; Turt, T. urticae; Iper, I. persulcatus; Isca, I, scapularis Data are derived from Oakeshott et al. (40), Reddy et al. (41), Xu et al. (42) and this study.

### 3.5 Genes of the *I. persulcatus* antioxidant system

To identify candidate antioxidant genes in *I. persulcatus*, we conducted homology-based searches using curated sequences from *Drosophila melanogaster*, *Anopheles gambiae*, and *Apis mellifera*—representative model insects and blood-feeding arthropods with well-annotated genomes. We identified forty putative antioxidant genes in the *I. persulcatus* transcriptome, a number comparable to those in *I. scapularis* (n = 41), *D. melanogaster* (n = 48), *A. gambiae* (n = 45), and *A. mellifera* (n = 35) (**Table 2** and **Supplementary Table S8**). The *I. persulcatus* antioxidant genes belong to nine antioxidant families: catalase (CAT), superoxide dismutase (SOD), thioredoxin peroxidase (TPX), glutathione peroxidase (GPX), heme-containing peroxidase (HPX), thioredoxin reductase (TRXR), methionine sulfoxide reductase (MSR), thioredoxin (TRX), and glutaredoxin (GRX). Some antioxidant gene families, such as CAT, GPX, MSR, TRX, and TRXR, were highly conserved across species (**Supplementary Figures S4**-**S7**). Additionally, phylogenetic analysis revealed that the HPX, SOD, and TPX families contained specific expanded genes, such as clade A of HPX (**Supplementary Figure S8**), *sod3* of SOD (**Supplementary Figure S9**), and *tpx7* of TPX (**Supplementary Figure S10**). Intriguingly, our phylogenetic analyses showed that some antioxidant genes of ixodid ticks were more closely related to those of vertebrates than insects. For example, *gpx5* of GPX clustered with its vertebrate orthologs (**Supplementary Figure S11**), and *sod3* of SOD formed two different monophyletic clades with insects and vertebrates (**Supplementary Figure S9**). In addition, the C-terminal motif of TRXR in ixodid ticks is composed of Gly-Cys-Sec-Gly (where Sec is selenocysteine) (**Supplementary Figure S7**), which is consistent with the sequence of mammalian species (43). By contrast, the selenocysteine residue has been replaced by a cysteine residue in insects (32).

**Table 2:** Summary of antioxidant systems in *I. persulcatus* and *I. scapularis*.

Antioxidant enzyme	Isca^a^	Iper^b^	Expression patterns of Iper			
			SE vs. UN^c^		EN vs. SE^d^	
			Up	Down	Up	Down
Primary antioxidant enzymes						
Superoxide dismutase	6	6	2	–	1	1
Catalase	1	1	1	–	–	–
Thioredoxin peroxidase	6	6	2	2	1	–
Glutathione peroxidase	3	3	–	–	–	1
Heme-containing peroxidase	13	8	4	1	–	–
Secondary antioxidant enzymes						
Thioredoxin reductase	1	1	–	–	–	–
Methionine sulfoxide reductase	2	4	–	1	–	1
Thioredoxin	5	6	–	3	2	1
Glutaredoxin	4	4	–	2	1	–

Iper, *I. persulcatus*; Isca, *I. scapularis*; UN, unfed tick; SE, semi-engorged tick; EN, fully engorged tick; ^a^,^b^ the number of antioxidant genes found in *I. scapularis* and *I. persulcatus*, respectively; ^c^,^d^ the number of significantly regulated antioxidant genes between semi-engorged and unfed ticks, fully engorged and semi-engorged ticks, respectively.

### 3.6 Transcript abundance patterns during different blood feeding phases

To investigate global gene expression changes during the blood-feeding process, three RNA-seq libraries for unfed, semi-engorged, and fully engorged female *I. persulcatus* ticks were constructed. After filtering low-quality reads from the raw data, the number of clean reads for each library were 10,361,442 (78.7%), 23,052,489 (85.0%), and 14,725,638 (77.9%), respectively (**Table 3**). The clean reads, ranging from 56.9% to 60.7% of each library were then mapped to the unigenes of the *I. persulcatus* tick. The number of unigenes with at least one mapped clean read was 42,089 in unfed ticks, 39,646 in semi-engorged ticks, and 37,337 in fully engorged ticks.

**Table 3:** Mapping summary.

Metric	Unfed tick	Semi-engorged tick	Fully engorged tick
Raw data	13,165,040	27,117,366	18,899,310
Clean reads	10,361,442	23,052,489	14,725,638
Clean reads/Raw data	78.70	85.01	77.92
Reads mapping to Unigenes	6,287,410	13,316,218	8,383,802
Reads mapping to Unigenes*	60.68	57.76	56.93
Unique mapped reads	5,052,810	10,284,067	6,150,391
Unique mapped reads†	80.36	77.23	73.36
Read-mapped Unigenes	42,089	39,646	37,337
Read-mapped Unigenes¶	73.97	69.68	65.62

*% of Clean reads; †% of Reads mapping to Unigenes; ¶% of Ref Unigenes.

**Figure 6:**
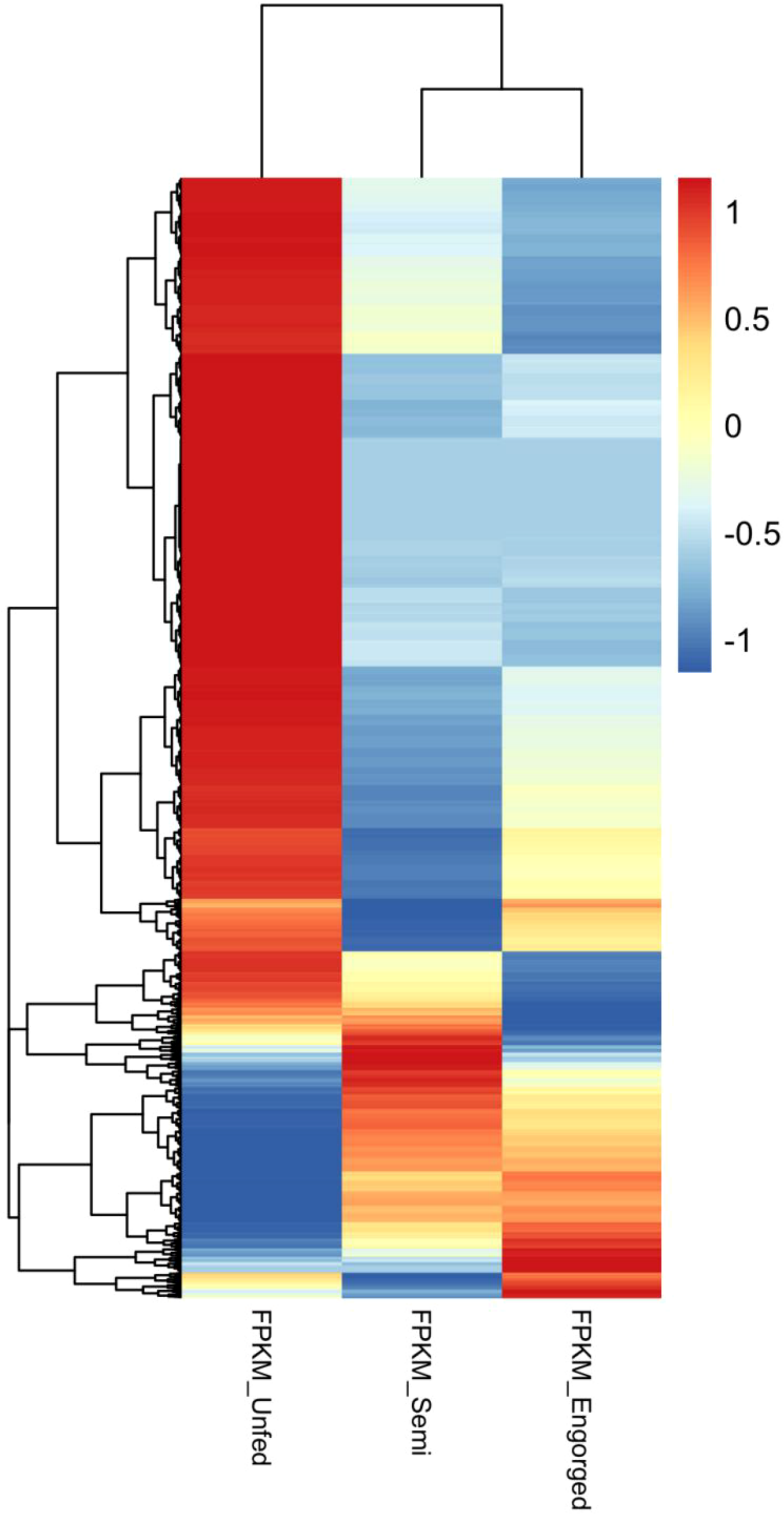
Heatmap of transcript abundance for differentially expressed unigenes across unfed, semi-engorged and fully engorged female *Ixodes persulcatus* ticks. Each row represents one differentially expressed unigene (defined as |log2 fold change| ≥ 1 and p < 0.005 in at least one pairwise comparison: semi-engorged vs. unfed or engorged vs. semi-engorged), and each column represents one feeding stage. FPKM values were transformed as log2(FPKM + 1) and standardized by row (z-score) prior to hierarchical clustering. Warmer and cooler colors indicate relatively higher and lower transcript abundance, respectively. Both genes and samples were clustered using Euclidean distance and complete linkage.

A descriptive comparison of transcript abundance revealed stage-specific shifts in gene expression (**Figure 6** and **Supplementary Table S9**). In the semi-engorged ticks, many of the highly abundant transcripts corresponded to genes associated with blood acquisition and digestion, including salivary gland proteins, cuticle proteins, secreted proteins, heme-binding proteins, transferrin receptors, and vitellogenins (**Supplementary Table S10**). These genes are known to participate in feeding, heme transport, and reproductive preparation. Conversely, transcripts with reduced abundance included those with unknown function or annotations related to salivary proteins and formin-like proteins.

In the transition to full engorgement, changes in transcript abundance were again observed (**Figure 6** and **Supplementary Tables S9**, **S10**). Among the annotated genes, highly abundant transcripts at this stage included those associated with cuticle expansion (*e.g.*, putative cuticle protein) and blood processing (*e.g.*, CLSP-8, secreted GGY protein). Notably, a unigene annotated as *Leishmania* spp. proteophosphoglycan ppg4 was strongly expressed in the fully engorged ticks, potentially suggesting the activation or persistence of *Leishmania*-related elements in late-stage feeding. Meanwhile, transcripts with decreased abundance included genes linked to immune responses (*e.g.*, PI3K, P450s, hemolectin) and muscle formation (*e.g.*, kettin).

Collectively, these transcriptomic shifts reveal a stage-specific reprogramming during the blood-feeding process, where semi-engorged ticks exhibit widespread down-regulation of transcripts compared to the unfed stage (**Figure 6**), while fully engorged ticks show transcriptional signatures associated with reproduction, cuticle expansion, and metabolic recovery.

### 3.7 Functional categories of transcripts with varying abundance across feeding phases

To explore functional trends across feeding phases, we conducted GO term annotation focusing on biological processes.

In semi-engorged ticks compared to the unfed stage, transcripts associated with blood acquisition, nutrient metabolism, respiratory processes, hormone synthesis, and egg development showed increased representation among GO terms (**Supplementary Table S11**). Immune-related processes were also prominently represented, possibly reflecting responses to microbial proliferation in the midgut. Additionally, transcripts involved in ROS detoxification were broadly distributed in enriched GO terms, with annotations corresponding to enzymes such as P450s, GSTs, GPXs, TPXs, SODs, HPXs, TRXs, dehydrogenases, and reductases. In contrast, GO terms associated with cell cycle, transcription, and translation were linked to transcripts less abundant in this stage, possibly reflecting energy allocation toward digestion and reproduction, consistent with previous observations in other tick species (44).

In fully engorged ticks compared to the semi-engorged stage, abundant transcripts were associated with metabolism, egg development, cuticle formation, immune response, and transcriptional regulation (**Supplementary Table S12**). Interestingly, some transcripts related to immune response and digestion were found across both enriched and reduced GO terms, suggesting dynamic regulation. Several detoxification-related transcripts, particularly those involved in oxidative stress responses, were less represented at this stage, potentially reflecting a reduction in ROS stress as digestion nears completion.

### 3.8 Expression patterns of detoxification and antioxidant genes

To characterize changes in detoxification and antioxidant systems, we examined the expression profiles of related genes across feeding stages in *I. persulcatus*.

Among the 166 annotated P450 genes, 66 exhibited noticeable differences in transcript abundance, including 57 transcripts most pronounced in semi-engorged ticks and 19 in fully engorged ticks. Many of these belonged to the CYP3 clan, which was the most expanded group within the P450 family (**Supplementary Table S5**). GSTs and CarEs displayed similar trends. For GSTs, 15 of 33 transcripts showed altered patterns, mostly from the Mu and Delta classes (**Supplementary Table S6**). For CarEs, 18 of 49 transcripts varied in abundance, predominantly within the expanded clade J (**Supplementary Table S7**). These patterns suggest a dynamic role of detoxification-related gene families during the feeding process.

Of the 40 antioxidant-related genes identified, 39 were detected in at least one feeding stage, and 33 were present across all stages, indicating consistent involvement in redox maintenance (**Supplementary Table S8**). Among them, 23 transcripts showed variable patterns, especially during the slow-feeding phase. For instance, catalase (CAT) increased in abundance, whereas hpx9 (a Duox/peroxidase component) decreased. These changes may reflect redox adaptation to different physiological demands during blood ingestion.

## 4 Discussion

### 4.1 Stage-specific transcriptomic remodeling during blood feeding

During off-host phases, ticks require a limited set of genes to support survival-related functions such as water collection and host seeking. Upon attachment and initiation of blood feeding, a broad transcriptomic reprogramming occurs. Genes related to off-host survival tend to decrease in activity, while those supporting blood ingestion, digestion, and tissue remodeling become more active (45, 46). This shift was reflected in our dataset, where transcript abundance generally declined in semi-engorged ticks compared to unfed ticks, suggesting a reallocation of resources in preparation for blood meal processing.

The semi-engorged ticks analyzed in this study were collected five days post-attachment, corresponding to the late slow-feeding phase—just before the onset of rapid engorgement. At this stage, transcripts associated with salivary gland remodeling, nutrient transport, and vitellogenesis began to accumulate, likely reflecting the physiological transition toward reproduction. Meanwhile, many transcripts related to host-seeking behavior and external defense were reduced in abundance, consistent with a shift in physiological priorities toward internal homeostasis.

Functional annotation and GO term enrichment revealed increased representation of genes involved in blood acquisition, nutrient metabolism, and cellular respiration. These trends align with the heightened metabolic demand during feeding. In parallel, transcripts associated with reactive oxygen species (ROS) detoxification were more prevalent, suggesting an oxidative burden imposed by blood intake. Heme and iron, released during hemoglobin degradation, are known to catalyze ROS generation, necessitating upregulation of antioxidant responses to maintain cellular integrity (11, 47). Conversely, transcripts associated with cell cycle progression, transcriptional activity, and protein biosynthesis were less abundant in the semi-engorged stage. This may reflect a physiological trade-off in which energy is redirected from cellular proliferation to stress mitigation and nutrient assimilation, as similarly observed in other blood-feeding arthropods (44).

In fully engorged ticks, transcriptome profiles revealed further remodeling. Increased abundance of transcripts linked to egg development, cuticle expansion, and late-stage metabolism suggests a transition into reproductive and post-feeding recovery phases. Meanwhile, reduced representation of immune- and ROS-related transcripts may indicate resolution of earlier oxidative stress. These trends are consistent with the declining physiological demands associated with blood digestion and the onset of post-feeding tissue restructuring (48).

It is worth noting that only 27.6% of the assembled unigenes were successfully annotated with GO terms. This relatively low annotation rate may be attributed to the limited availability of tick-specific reference genomes and transcriptomic datasets in public databases. In addition, some unigenes may represent non-coding RNAs, highly divergent sequences, or novel transcripts without known homologs. While this limitation constrains the scope of functional interpretation, the annotated subset still provides valuable insights into key biological processes relevant to blood feeding and redox homeostasis.

### 4.2 Expansion of detoxification and antioxidant gene families

Our comparative genomic analysis across seven tick species and one mosquito outgroup provides an evolutionary framework for interpreting the detoxification and antioxidant repertoires observed in *I. persulcatus*. An UpSet analysis of orthogroup presence–absence patterns further showed that these genomes share a large core of gene families present in all eight species, likely corresponding to conserved arthropod housekeeping functions, alongside tick-restricted and *I. persulcatus*-specific orthogroups (**Figure 4**). These patterns indicate that tick genomes combine a conserved arthropod backbone with lineage-specific innovations, some of which are plausibly associated with hematophagy, host interaction, and vector competence.

Our transcriptomic analysis revealed that *I. persulcatus* possesses species-specific expansions in major detoxification gene families. The CYP2 and CYP3 clans—typically involved in hormone metabolism and xenobiotic detoxification, respectively—were notably expanded relative to insects, supporting a potential enhancement in metabolic detoxification capacity (29). Within the GST superfamily, Mu and Delta classes were also expanded. The Mu class, previously thought to be vertebrate-specific, has now been identified in several Acari species (6, 30, 42), and may play a conserved role in managing heme toxicity. Functional studies from other tick species support this view. For example, Zhao et al. (49) demonstrated that HrGSTm1, a Mu-class GST from Hyalomma rufipes, is not only differentially expressed across tissues and developmental stages, but also shows strong antioxidant activity *in vitro* and impacts tick physiology when knocked down via RNA interference. These results support a broader role for Mu GSTs in oxidative stress mitigation and detoxification during blood feeding. The total number of detoxification genes in *I. persulcatus* is comparable to that in hematophagous mosquitoes, which also show expansion in these families compared to non-blood-feeding insects (50). These lineage-specific expansions likely reflect an evolutionary adaptation to cope with the physiological burdens of blood feeding, especially the need to neutralize heme toxicity, reactive oxygen species, and host-derived molecules.

In parallel, the antioxidant system in *I. persulcatus* also exhibited complexity and expansion. Blood feeding imposes oxidative stress via host-derived ROS, heme digestion, and pathogen colonization. Ticks rely on an elaborate antioxidant defense to mitigate such stress and maintain redox balance. We identified 40 antioxidant genes, including expanded clades such as HPX clade A, *sod3*, and *tpx7*, suggesting that antioxidant genes have undergone duplication and divergence to support stage- and tissue-specific demands. Interestingly, some *I. persulcatus* antioxidant genes—such as *gpx5, sod3*, and TRXR containing a selenocysteine motif—show phylogenetic similarity to vertebrate orthologs, suggesting an evolutionary trajectory distinct from that in insects. This divergence may reflect adaptation to vertebrate-derived oxidative environments and is further supported by differences in GST family composition between *I. persulcatus* and *I. scapularis* (41). These insights point toward a possible convergence with vertebrate antioxidant strategies or a unique lineage-specific evolution in ticks.

Together, the observed expansion and differential expression of detoxification and antioxidant gene families underscore their essential role in physiological adaptation during tick hematophagy. These gene families likely help ticks neutralize toxic byproducts, maintain midgut tissue integrity, and support reproductive success following engorgement.

### 4.3 Temporal regulation of detoxification and antioxidant genes during blood feeding

The expression dynamics of detoxification and antioxidant gene families highlight their central roles in the physiological adaptation of ticks to blood feeding. Notably, genes from expanded lineages—such as CYP3 clan P450s, GSTs, and CarEs—were differentially expressed across feeding stages, suggesting that hematophagy imposes distinct and temporally shifting metabolic and oxidative challenges. These shifts likely reflect changing physiological demands, such as detoxifying host-derived xenobiotics, heme, and immune effectors.

For the antioxidant system, most genes were expressed constitutively, consistent with a basal role in redox homeostasis. However, the slow-feeding phase appears to be the critical window for oxidative stress responses, evidenced by the surge in differential gene expression, especially CAT upregulation and hpx9 downregulation. While previous studies reported Duox/peroxidase upregulation after blood feeding (51), our data suggest that their expression may be transient and stage-dependent, possibly suppressed during late feeding. Such discrepancies likely stem from differences in sampling time points and tissue specificity.

Our whole-body transcriptomic approach, while comprehensive, may have masked organ-specific expression signatures. For instance, Sabadin et al. (52) reported that in *Rhipicephalus microplus* female ticks, most antioxidant and detoxification enzymes exhibit higher expression and enzymatic activity in the ovaries and fat body during the partially engorged stage. In contrast, gene expression patterns in the midgut were more variable, with distinct shifts in enzyme activity observed between feeding stages. Similarly, in *Haemaphysalis longicornis*, the expression of *HlPrx2* is significantly upregulated during blood feeding across multiple tissues (53).

These findings, together with our results in *I. persulcatus*, support the notion that oxidative stress responses are finely tuned at both the temporal and tissue levels. The dynamic expression of detoxification and antioxidant genes likely represents a coordinated physiological strategy to manage the progressive accumulation of heme, iron, and ROS during the blood meal, ultimately ensuring tissue protection, midgut integrity, and reproductive fitness.

### 4.4 Putative *Leishmania*-like sequences in *I. persulcatus*

Our detection of Leishmania-like transcripts in *I. persulcatus* aligns with a growing body of research exploring the possible involvement of ticks in *Leishmania* transmission. Although sand flies remain the only confirmed biological vectors, studies have increasingly reported *Leishmania* DNA in tick species. For example, Rojas-Jaimes et al. (54) detected high loads of *Leishmania* DNA in *R. microplus* and *A. sabanerae* collected from wild mammals in the Peruvian Amazon, with positivity rates exceeding 90%, suggesting possible acquisition via blood feeding. However, the study did not assess tissue localization or parasite development. In contrast, Viol et al. (55) provided more direct evidence by detecting *Leishmania* promastigotes—the parasite’s infective stage—in the intestines, ovaries, and salivary glands of *R. sanguineus* ticks using both IHC and RT-PCR, strongly suggesting the potential for partial development within the tick host. Nevertheless, vector competence remains unproven without evidence of successful transmission. Other studies have similarly detected *Leishmania* DNA in questing ticks. For instance, Magri et al. (56) reported *Leishmania* kDNA in unfed larvae, nymphs, and adult *Ixodes ricinus* in Italy, supporting possible transstadial or transovarial persistence. However, such molecular detections may reflect environmental contamination or ingested host material, and do not confirm vectorial capacity (57).

In our case, we detected a small set of unigenes whose top BLAST hits matched genes annotated to *Leishmania* spp., most of which were homologs of proteophosphoglycans implicated in parasite colonization, transmission, and mammalian infection. However, these hits were not consistently supported across multiple reference databases (e.g., NT, SwissProt), and the annotation confidence is therefore limited (**Supplementary Table S13**). We therefore interpret these sequences with caution and make no claims about *I. persulcatus* as a vector. Future studies should incorporate tissue localization, life-stage identification, and experimental transmission assays to rigorously assess tick involvement in *Leishmania* ecology.

## 5 Conclusions

*I. persulcatus* is the predominant tick species in Northeastern China, and is a major vector involved in the transmission of tick-borne diseases. In this study, we performed a comprehensive transcriptomic analysis of *I. persulcatus* across three critical blood-feeding stages—unfed, semi-engorged, and fully engorged—and generated a high-quality reference transcriptome comprising 56,900 unigenes. Functional annotation and comparative analyses revealed species-specific expansions in key detoxification and antioxidant gene families, including cytochrome P450s, glutathione S-transferases, and carboxylesterases. These genes showed dynamic and stage-dependent expression patterns, reflecting their roles in xenobiotic clearance, redox homeostasis, and physiological adaptation during blood feeding.

Stage-associated transcriptomic trends further indicated dynamic regulation of metabolism, respiration, hormone synthesis, reproduction, immune modulation, and oxidative stress responses throughout feeding progression. These observations highlight the complex molecular mechanisms that enable ticks to cope with blood meal–induced physiological challenges.

In summary, these findings provide novel insights into the molecular physiology of *I. persulcatus*, and establish a foundational dataset for investigating tick-pathogen interactions. Our work offers valuable targets for vector control strategies and pathogen monitoring, with implications for mitigating the public health burden posed by tick-borne diseases.

## Data Availability

The datasets presented in this study can be found in online repositories. The names of the repository/repositories and accession number(s) can be found in the article/**Supplementary Material**.
