# Supplementary material for: Blood feeding-induced transcriptomic changes in the hard tick Ixodes persulcatus
Source: Front Insect Sci. 2026 Feb 23;6:1669026. doi: 10.3389/finsc.2026.1669026 (PMC12968816; doi:10.3389/finsc.2026.1669026)
Supplement: Supplementary file 14 [file SupplementaryFile1.docx]

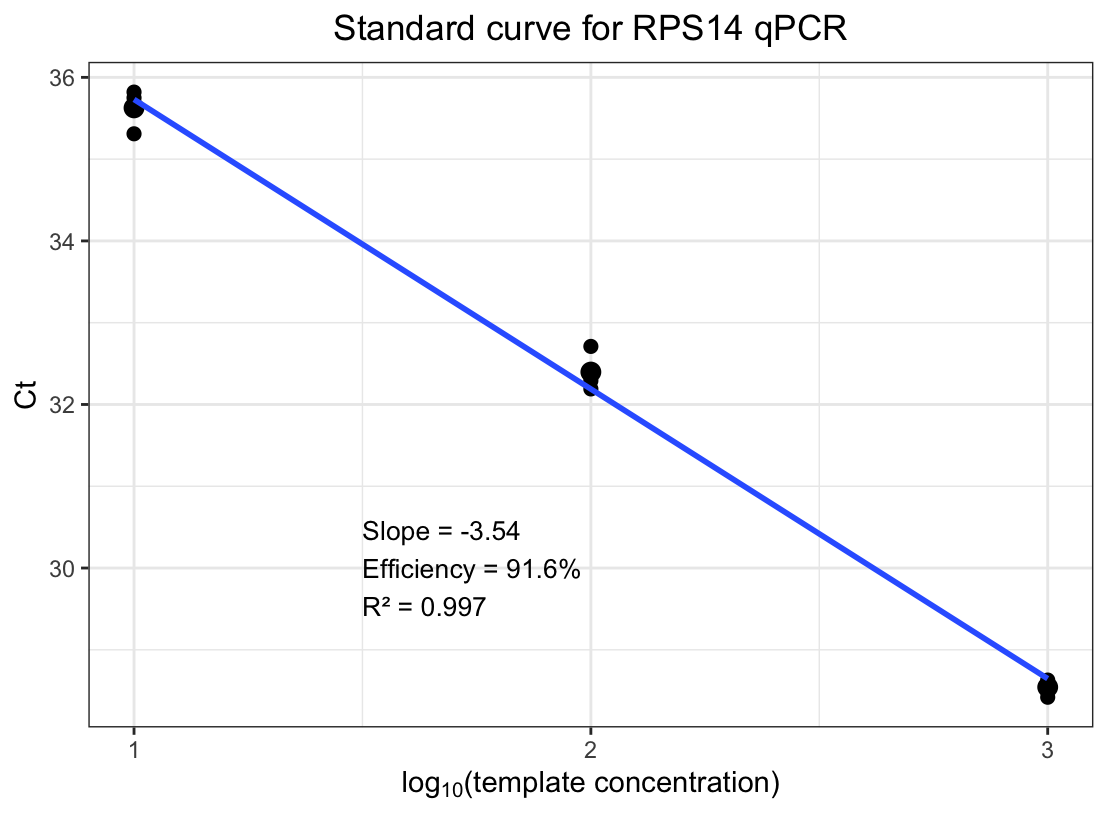


**Fig. S1.** Standard curve for the *RPS14* qPCR assay. A three-point 10-fold dilution series of cDNA was tested in technical triplicates, and Ct values were plotted against the log10-transformed template concentration. Each small dot represents an individual technical replicate, and larger symbols indicate the mean Ct for each dilution. The linear regression fitted to the mean Ct values yielded a slope of −3.54, corresponding to an amplification efficiency of 91.6%, with an R² of 0.997.


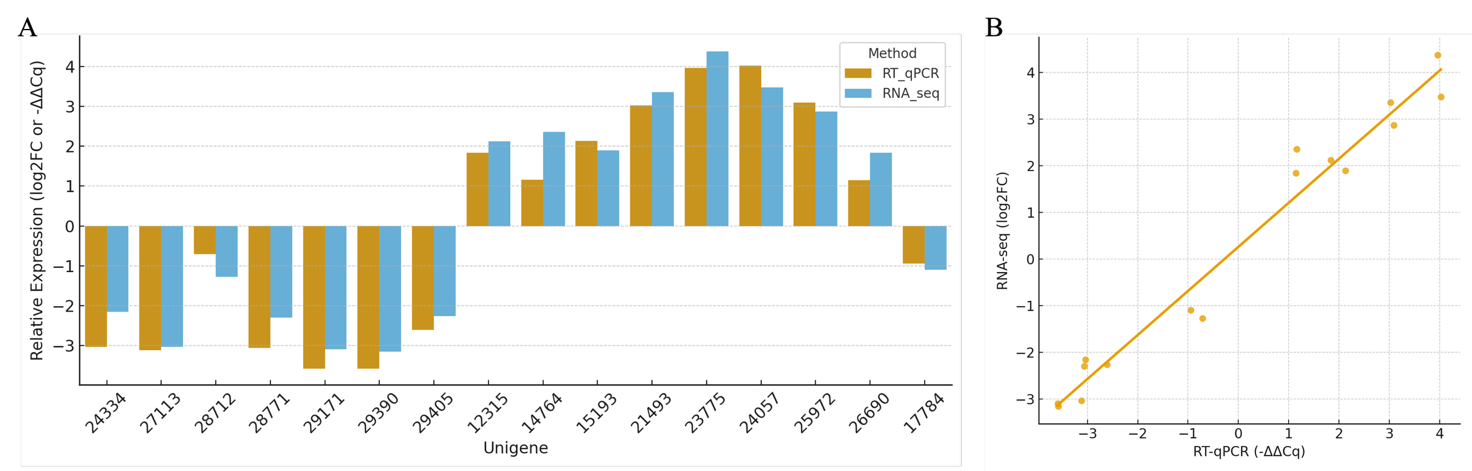


**Fig. S2.** Validation of RNA-seq results by RT-qPCR.

(A) Relative expression levels of 16 selected unigenes measured by RT-qPCR across different feeding stages of Ixodes persulcatus. Gene expression was normalized to reference genes and calculated using the 2^–ΔΔCt method. (B) Correlation analysis between RNA-seq and RT-qPCR results (log₂ fold change). A strong positive correlation (Pearson’s r = 0.987, p < 0.0001) indicates high consistency between the two datasets.


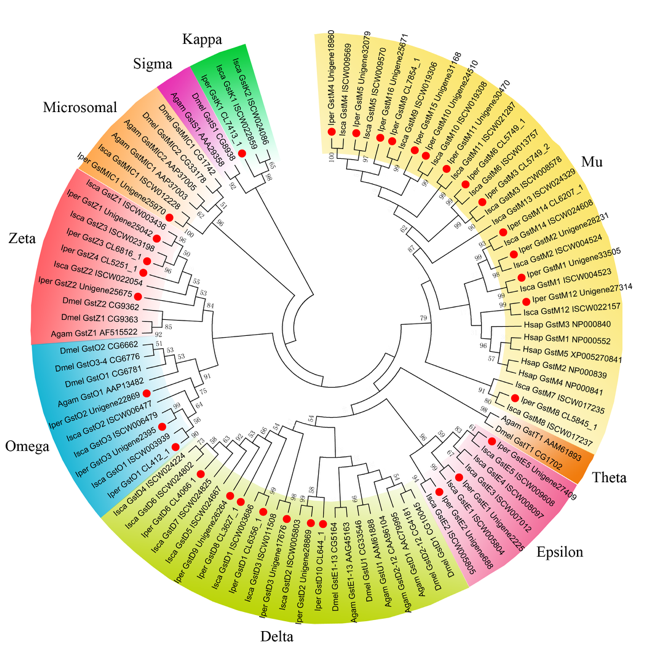


**Fig. S3.** Maximum likelihood tree of GST family. The putative members of GST family in *I. persulcatus* were marked with red dots. In total, 33 GST genes of *I. persulcatus* were clustered as seven different classes, namely Mu (14), Delta (7), Zeta (4), Epsilon (3), Omega (3), Kappa (1) and microsomal (1). Bootstrap value (1,000 replicates) ≥50% are shown. Agam, for *A. gambiae*; Amel, for *A. mellifera*; Dmel, for *D. melanogaster*; Hsap, *H. sapiens*; Iper, for *I. persulcatus*; Isca, for *I. scapularis*.


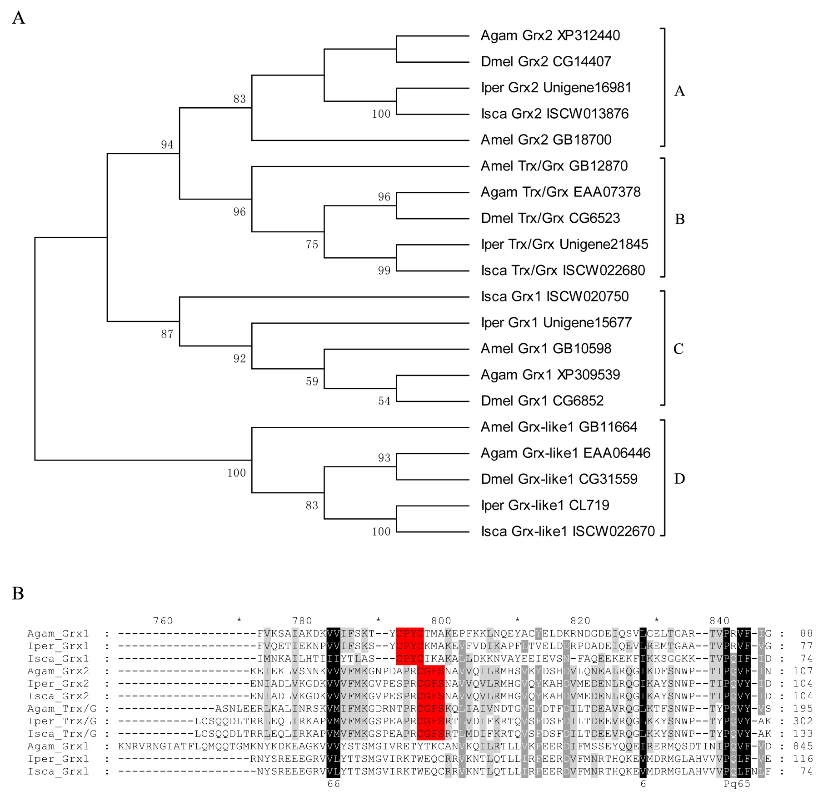


**Fig. S4.** Phylogenetic analysis and sequence alignment of GRX family. (A) Maximum likelihood tree of GRX family. Each entry has a species name (Agam, for *A. gambiae*; Amel, for *A. mellifera*; Dmel, for *D. melanogaster*; Iper, for *I. persulcatus*; Isca, for *I. scapularis* ). Bootstrap value (1,000 replicates) ≥50% are shown. (B) Fragment of an alignment of GRX family proteins. The conserved active sites are highlighted.

**
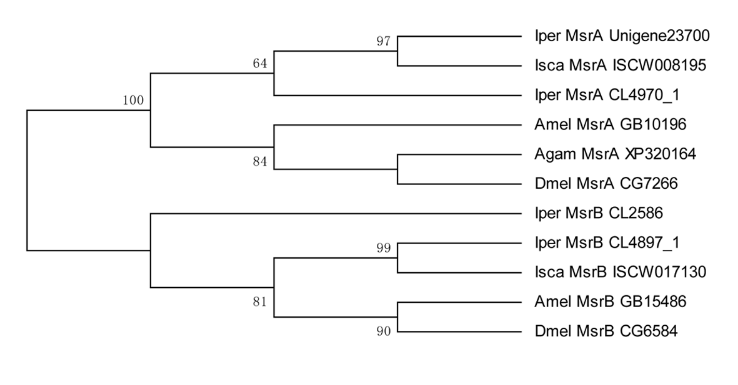
**

**Fig. S5.** Maximum likelihood tree of MSR family. Bootstrap value (1,000 replicates) ≥50% are shown. Agam, for *A. gambiae*; Amel, for *A. mellifera*; Dmel, for *D. melanogaster*; Iper, for *I. persulcatus*; Isca, for *I. scapularis*.

**
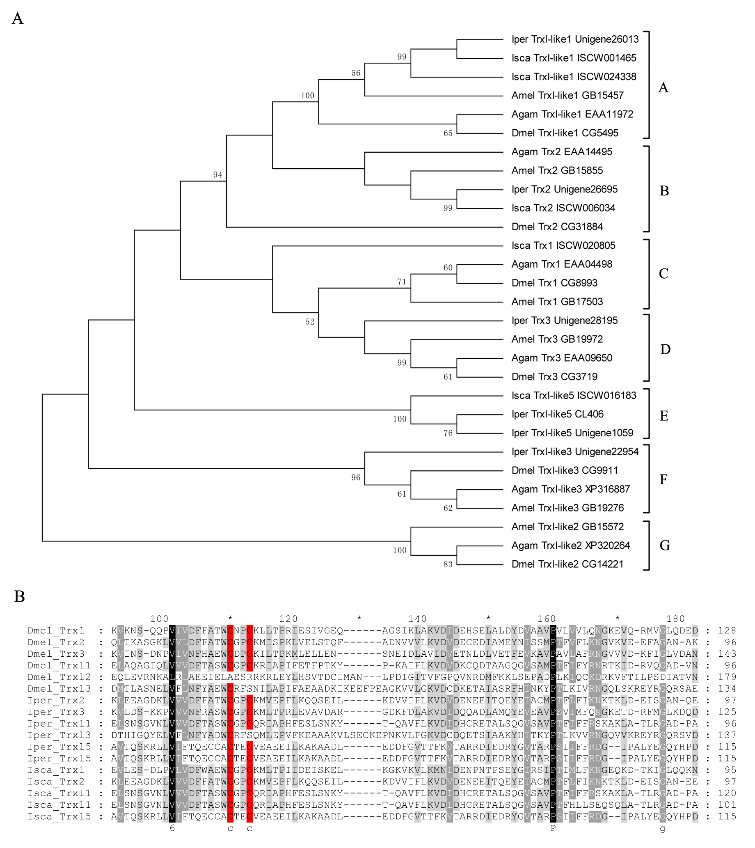
**

**Fig. S6**. Phylogenetic analysis and sequence alignment of TRX family. (A) Maximum likelihood tree of TRX family. Each entry has a species name (Agam, for *A. gambiae*; Amel, for *A. mellifera*; Dmel, for *D. melanogaster*; Iper, for *I. persulcatus*; Isca, for *I. scapularis* ). Bootstrap value (1,000 replicates) ≥50% are shown. (B) Fragment of an alignment of TRX family proteins. The conserved active sites are highlighted.

**
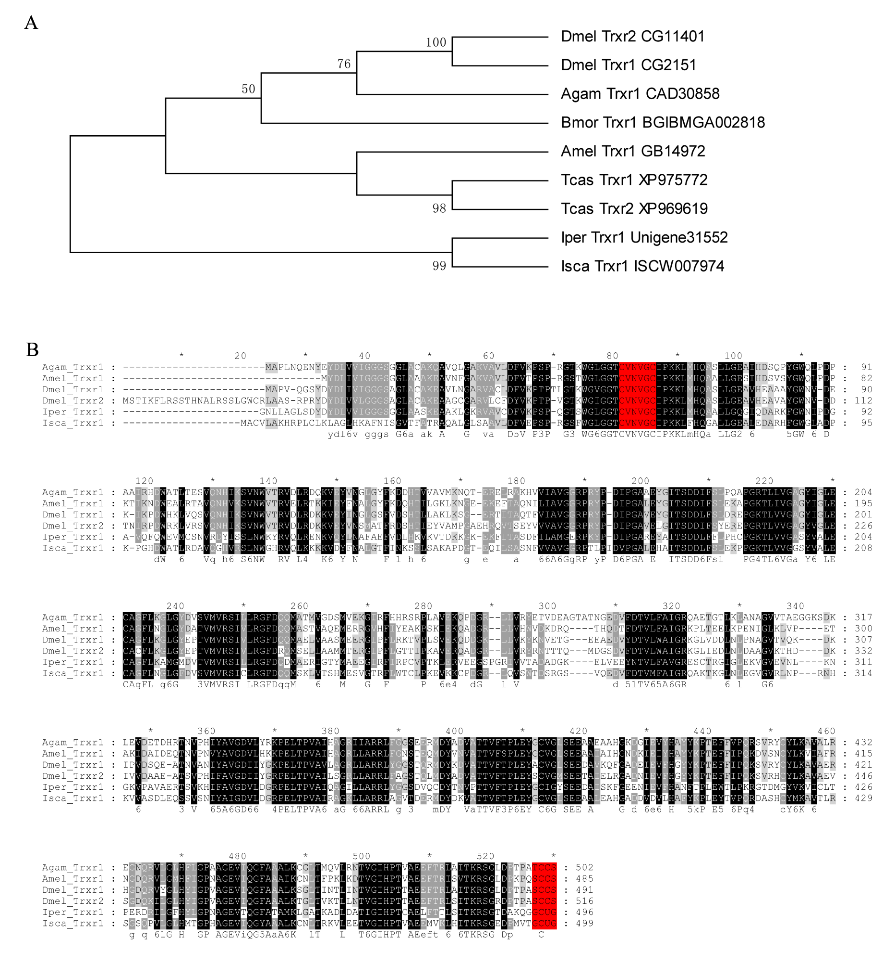
**

**Fig. S7.** Phylogenetic analysis and sequence alignment of TRXR family. (A) Maximum likelihood tree of TRXR family. Each entry has a species name (Agam, for *A. gambiae*; Amel, for *A. mellifera*; Dmel, for *D. melanogaster*; Iper, for *I. persulcatus*; Isca, for *I. scapularis* ). Bootstrap value (1,000 replicates) ≥50% are shown. (B) Alignment of TRXR family proteins. The sequence of redox-active centers are highlighted. The C-terminal motif of TRXR in ixodid ticks is composed of Gly-Cys-Sec-Gly (where Sec is selenocysteine). However, the selenocysteine residue has been replaced by a cysteine residue in insects.

**
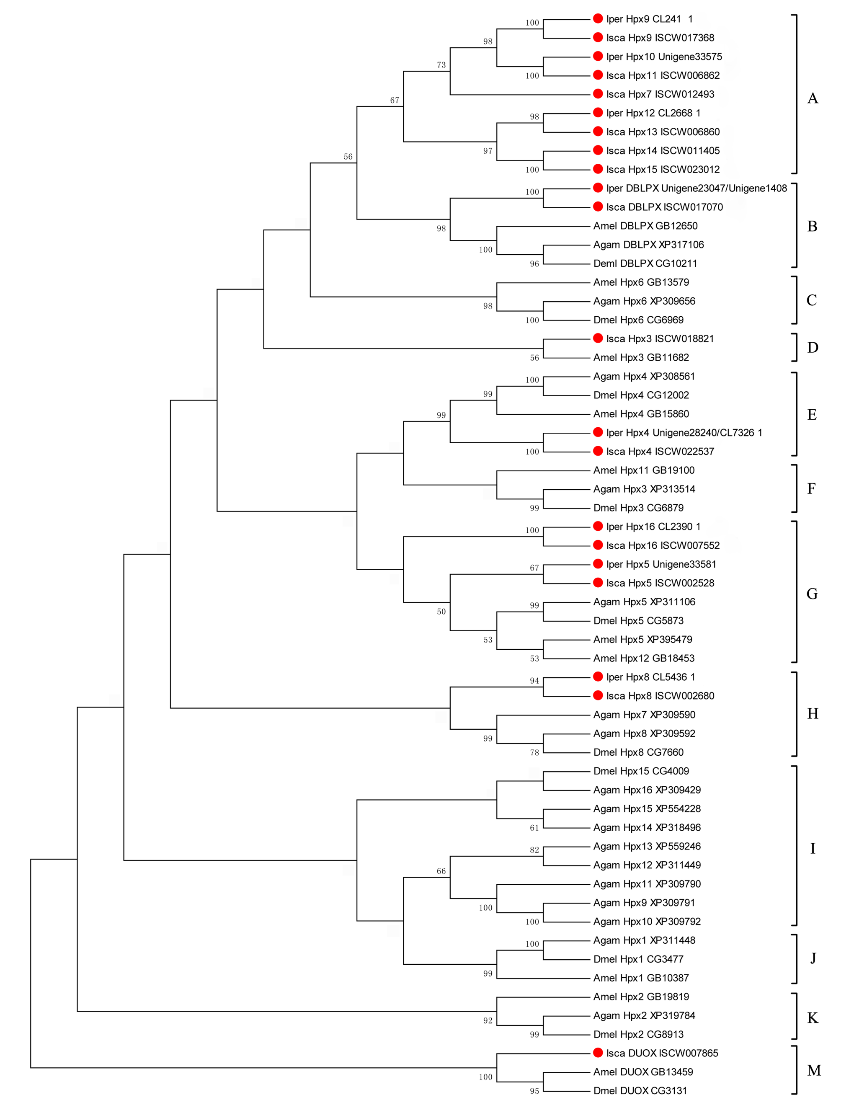
**

**Fig. S8.** Maximum likelihood tree of HPX family. The putative HPX members of *I. persulcatus* and *I. scapularis* were marked with red dots. It’s obvious that the ixodid HPX genes in clade A experienced specific expansions. Bootstrap value (1,000 replicates) ≥50% are shown. Agam, for *A. gambiae*; Amel, for *A. mellifera*; Dmel, for *D. melanogaster*; Iper, for *I. persulcatus*; Isca, for *I. scapularis*.

**
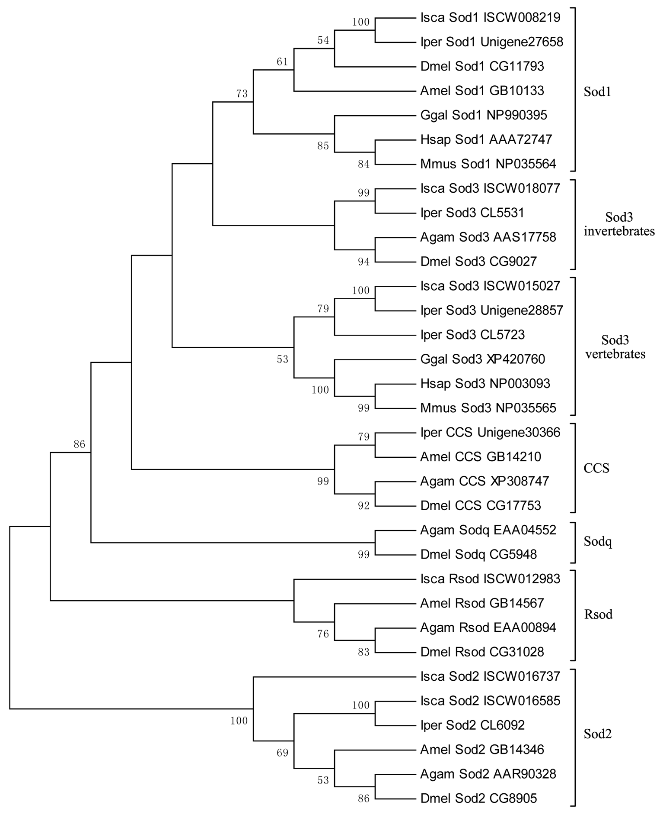
**

**Fig. S9.** Maximum likelihood tree of SOD family. Bootstrap value (1,000 replicates) ≥50% are shown. Agam, for *A. gambiae*; Amel, for *A. mellifera*; Dmel, for *D. melanogaster*; Ggal, *G. gallus*; Hsap, *H. sapiens*; Mmus, *M. musculus*; Iper, for *I. persulcatus*; Isca, for *I. scapularis*.

**
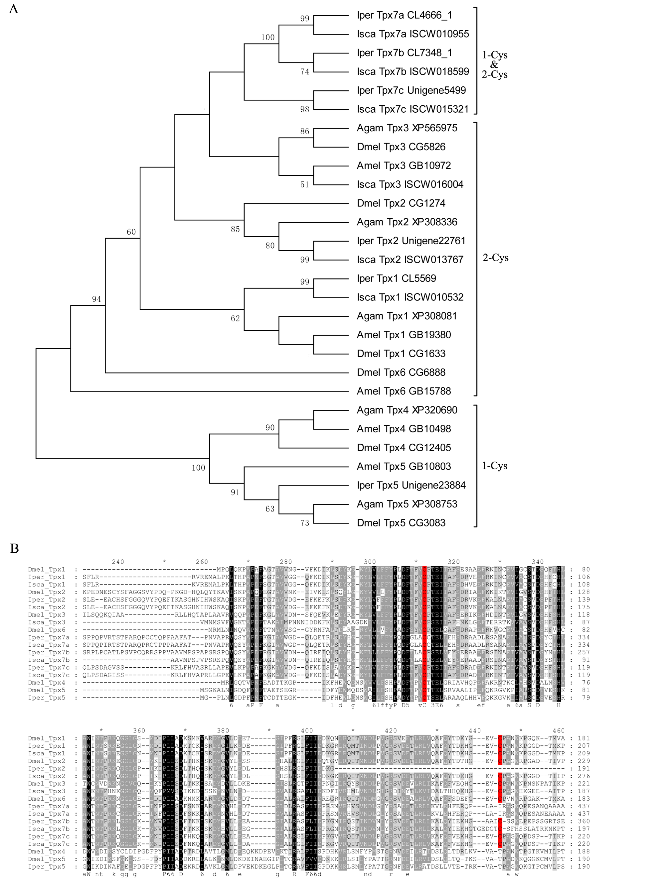
**

**Fig. S10.** Phylogenetic analysis and sequence alignment of TPX family. (A) Maximum likelihood tree of TPX family. The phylogenetic analysis showed that the putative members of TPX family were classed into three subfamilies: 1-Cys, 2-Cys and a novel subfamily including both 1-Cys and 2-Cys. Bootstrap value (1,000 replicates) ≥50% are shown. Agam, for *A. gambiae*; Amel, for *A. mellifera*; Dmel, for *D. melanogaster*; Iper, for *I. persulcatus*; Isca, for *I. scapularis*. (B) Fragment of an alignment of TPX family proteins. Amino acids of the catalytic site are highlighted.

**
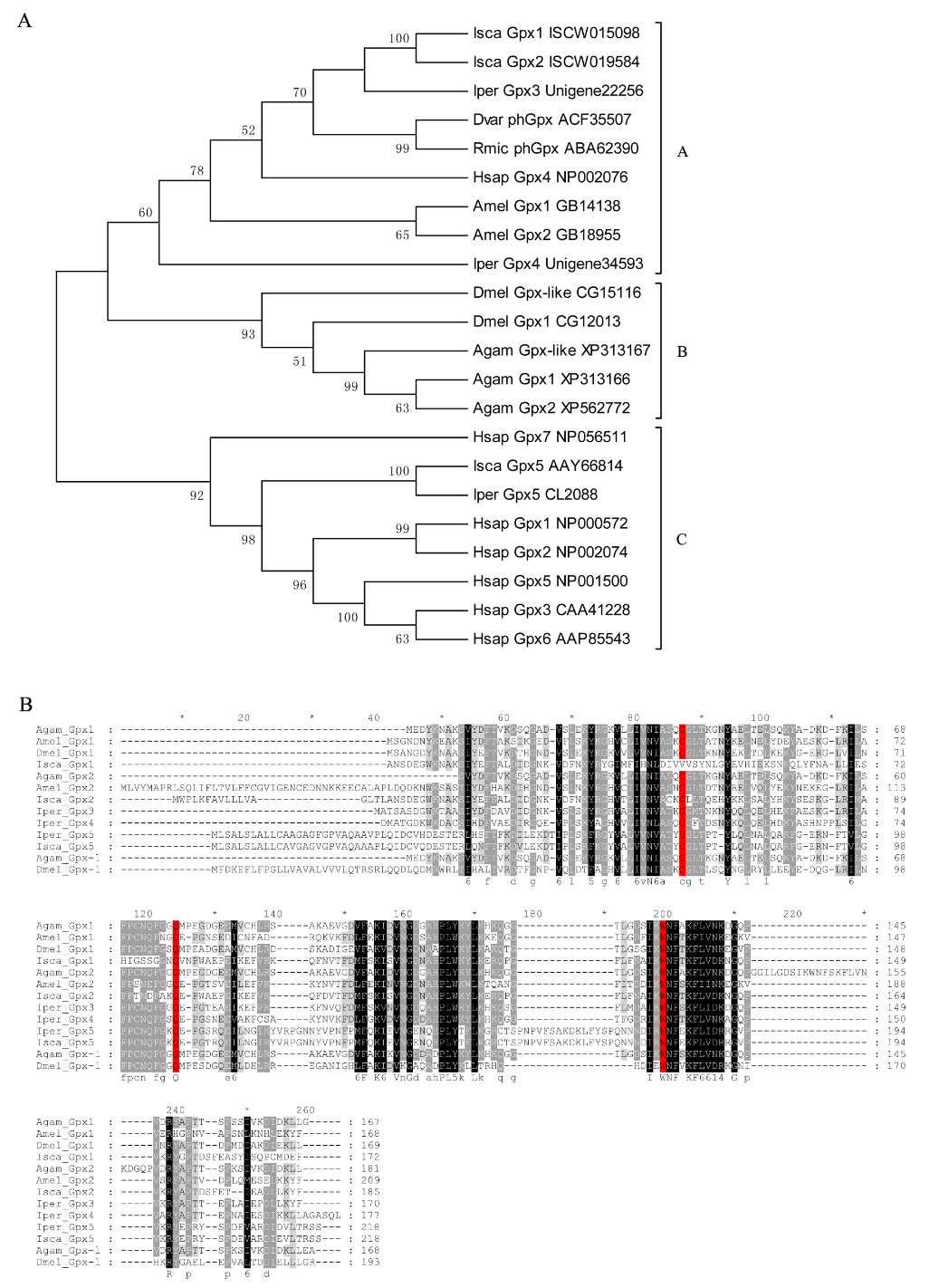
**

**Fig. S11.** Phylogenetic analysis and sequence alignment of GPX family. (A) Maximum likelihood tree of GPX family. Each entry has a species name (Agam, for *A. gambiae*; Amel, for *A. mellifera*; Dmel, for *D. melanogaster*; Dvar, *D. variabilis*; Hsap, *H. sapiens*; Iper, for *I. persulcatus*; Isca, for *I. scapularis*; Rmic, *R. microplus*). Bootstrap value (1,000 replicates) ≥50% are shown. (B) Alignment of GPX family proteins. Amino acids of the catalytic site are highlighted.
